# From Atmosphere to Health Outcomes: Analyzing Predictors of Respiratory Disease Mortality in Turkiye

**DOI:** 10.1002/puh2.70003

**Published:** 2024-09-18

**Authors:** Mehmet Kocak, Asli Nurefsan Kocak

**Affiliations:** ^1^ International School of Medicine, Istanbul Medipol University Beykoz Istanbul Turkey

**Keywords:** asthma, COPD, longitudinal study, mortality, pneumonia, predictors, public health, Turkiye

## Abstract

**Background:**

Asthma, chronic obstructive pulmonary disease (COPD), and pneumonia are significant contributors to morbidity and mortality worldwide. Identifying the predictors of mortality due to these diseases is crucial for effective public health interventions.

**Methods:**

We conducted a longitudinal trajectory modeling using SAS TRAJ procedures on data from 81 provinces in Turkiye, categorizing death rates into two profiles for asthma and COPD and three for pneumonia. Environmental and socioeconomic factors were examined as potential predictors through logistic regression modeling.

**Results:**

For asthma, none of the predictors met the false discovery rate (FDR) threshold for significance, suggesting the need for further research. In contrast, COPD predictors showed robust associations with mortality rates, particularly concerning environmental factors such as humidity and temperature. Pneumonia mortality was significantly associated with factors, including air pressure, humidity, temperature, alcohol use, and particulate matter.

**Conclusion:**

The study reveals distinct mortality profiles for respiratory diseases and highlights the importance of environmental and lifestyle factors as predictors. These findings emphasize the need for targeted public health strategies and interventions to manage these diseases effectively.

## Graphical abstract

Asthma, COPD, and pneumonia are significant contributors to morbidity and mortality worldwide. We have shown that provinces with increased air pressure and humidity report increased respiratory deaths, whereas provinces with higher temperature or higher variation of environmental factors report reduced respiratory deaths. Therefore, regionally targeted public health strategies must be developed.

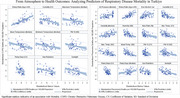

## 1 Introduction

Respiratory diseases (RD), such as asthma, chronic obstructive pulmonary disease (COPD), and pneumonia, continue to be major health challenges globally, with their impact and prevalence varying significantly across different regions. The burden of these diseases is not only a matter of health but also has substantial socioeconomic implications.

In Africa, RD are often compounded by factors like air pollution and infectious diseases, with tuberculosis and respiratory infections being major concerns alongside asthma and COPD [1, 2]. The challenges of managing these diseases in Africa are highlighted by the limited evidence on pulmonary rehabilitation and the efficacy of treatments in sub‐Saharan Africa [3]. Additionally, air pollution related to traffic is a significant contributor to chronic RD in the continent [4].

Europe faces a dual challenge: managing the high prevalence of COPD and asthma, particularly in Eastern Europe due to smoking and air pollution, and addressing these conditions in an aging population. The global burden of chronic RD, including their economic impact, is a significant concern, with variations observed among Western, Central, and Eastern Europe [5]. The trends in prevalence and incidence of chronic RD from 1990 to 2017 further illustrate the evolving landscape of these diseases in Europe and other regions [6].

The Americas present a diverse picture, with North America experiencing higher prevalence and mortality rates due to lifestyle factors and air quality, whereas South America grapples with disparities in healthcare access and environmental factors affecting disease management. In Asia, the burden of RD is heavily influenced by urban air pollution, smoking, and occupational exposures, making COPD and pneumonia particularly prevalent.

Australia, with its unique geographical and demographic characteristics, faces challenges in rural health service access and higher rates of asthma, especially among indigenous populations [7, 8]. A global review of asthma triggers across six continents, including Africa, Asia, Australia, Europe, North America, and South America, reveals commonalities in triggers such as allergens, tobacco smoke, and air pollutants [9].

The World Health Organization's report on the top 10 causes of death globally as of 2020 includes chronic RD like COPD and asthma, underscoring their significance in global health [10]. Yiğit and Yiğit [11] reported that COPD was the 11th leading cause of death in 1990 in Turkiye, which became the 7th leading cause of death in 2019. Yorgancıoglu et al. [12] reported that asthma is within the top 20 leading causes of death with asthma prevalence between 2% and 5% among adults. Pneumonia as well remains a critical health issue, particularly among the elderly and individuals with comorbid conditions. The incidence of pneumonia in Turkiye has been rising, with significant seasonal variations. A study by Yildiz et al. [13] indicates that the incidence of pneumonia peaks during the winter months, coinciding with higher rates of respiratory infections [13]. The study emphasizes the importance of vaccination, especially pneumococcal and influenza vaccines, in preventing pneumonia‐related complications and reducing hospital admissions.

In general, the global trends in RD are not only influenced by advancements in healthcare and changes in lifestyle but are also significantly impacted by environmental factors. Air pollution, climate change, and socioeconomic–environmental interactions represent key areas where public health interventions can make a substantial difference. Addressing these environmental determinants is crucial for reducing the global burden of RD. In this study, we aim to describe the longitudinal change of deaths due to RD in Turkiye at province level and to investigate the impact of environmental markers on RD death profiles.

## 2 Methods

### 2.1 Data Sources

Turkish Statistical Institute publishes the mortaliy counts by the primary disease areas overall and for each of the 81 provinces of Turkiye. We requested the specific mortality data for asthma, obstructive pulmonary disease (COPD), and pneumonia for years 2010–2019. We also obtained the population sizes of each province in these years and expressed the province‐level, disease‐specific death rates as the number of deaths per 100,000 population.

Similarly, Turkish Ministry of Environment and Urbanization reports key environmental markers for each province annually. We requested the environmental and meteorological domain of our data, including the monthly average measurements of particulate matter 10 and 2.5 (PM_10_ and PM_2.5_), sulfur dioxide (SO_2_), carbon monoxide (CO), nitrogen dioxide (NO_2_), and ozone (O_3_), air pressure, humidity, rainy days in a year, maximum‐average‐minimum temperatures, wind speed as well as total sunlight, sun radiation, and electromagnetic field. For the environmental and meteorological markers, our primary representation was the annual averages; however, we also used the annual standard deviation (SD) over time and coefficient of variation (CV) as other markers of environmental variation within province as well.

We also obtained the elderly age population (age 65+) and male ratio from the Turkish Statistical Institute for each province and the province‐level smoking, alcohol consumption, and exposure to second‐hand smoke rates from national surveys to represent the behavioral domain of our data included.

### 2.2 Statistical Analysis

Using the TRAJ procedure developed by Jones, Nagin, and Roeder [14], we determined the ideal number of change‐profiles on the basis of the goodness of fit statistics. For asthma and COPD, 2 change‐profiles emerged, whereas for pneumonia, there were 3 change‐patterns that better distinguished 81 provinces.

For asthma and COPD, we constructed logistic regression models to investigate the association of environmental and behavioral data with the likelihood of higher asthma and COPD profiles. For pneumonia, we employed an ordinal logistic regression approach where the odds of being in a higher ordinal category of pneumonia profiles for each unit‐increase in a given predictor were modeled.

As we considered a total of 52 predictors, to address multiplicity, we used the false discovery rate (FDR) approach by Benjamini and Hochberg [15] in determining a list of potential predictors for further investigations. To illustrate the proposed associations graphically, we presented the scatter plots using the standardized measures of both the response variable and the predictors. All analyses were conducted using SAS (R) Version 9.4 (Cary, NC, USA).

## 3 Results

**FIGURE 1:**
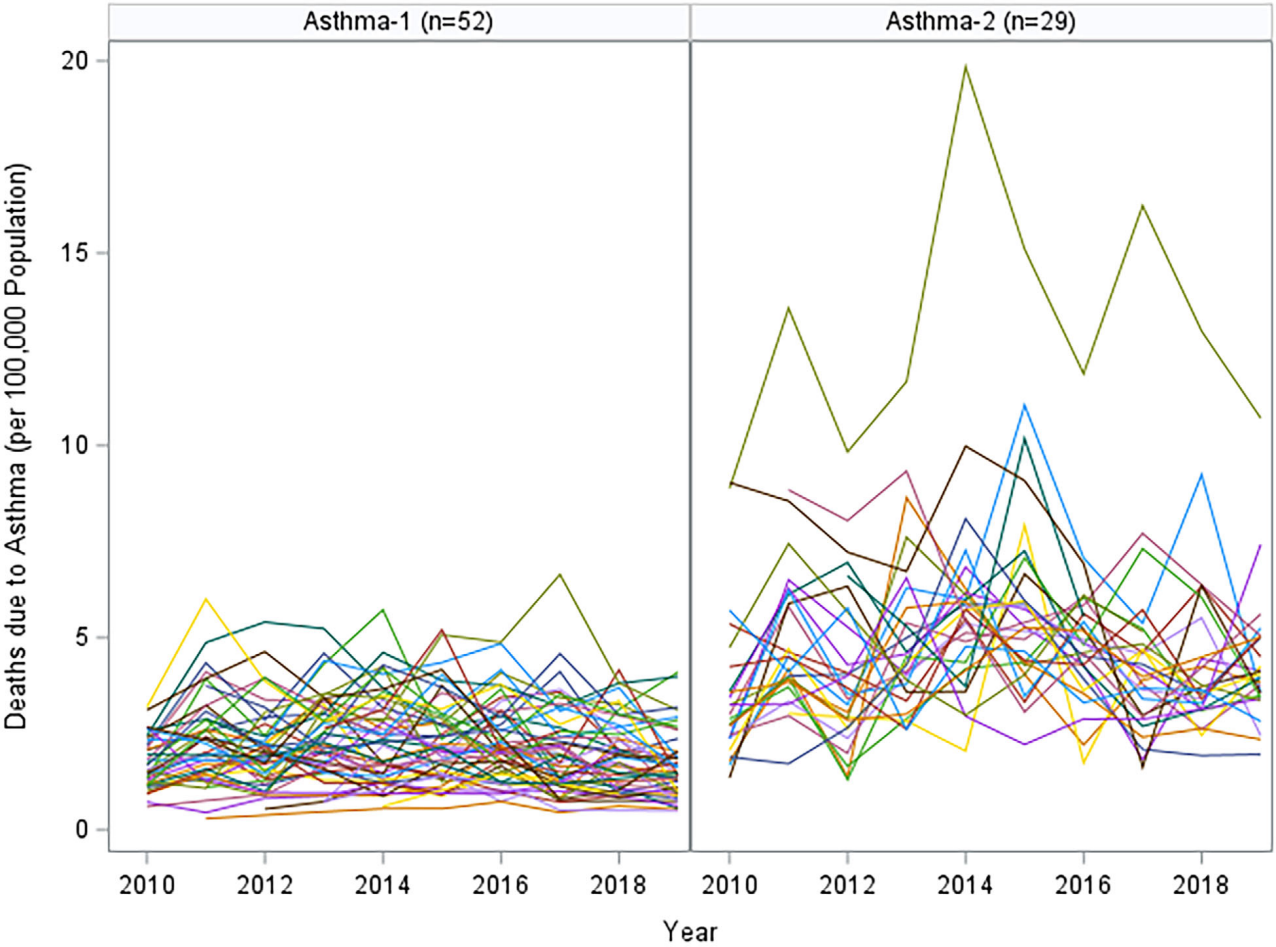
Asthma profiles by SAS TRAJ procedure (each line represents the change profile over 10 years).

**FIGURE 2:**
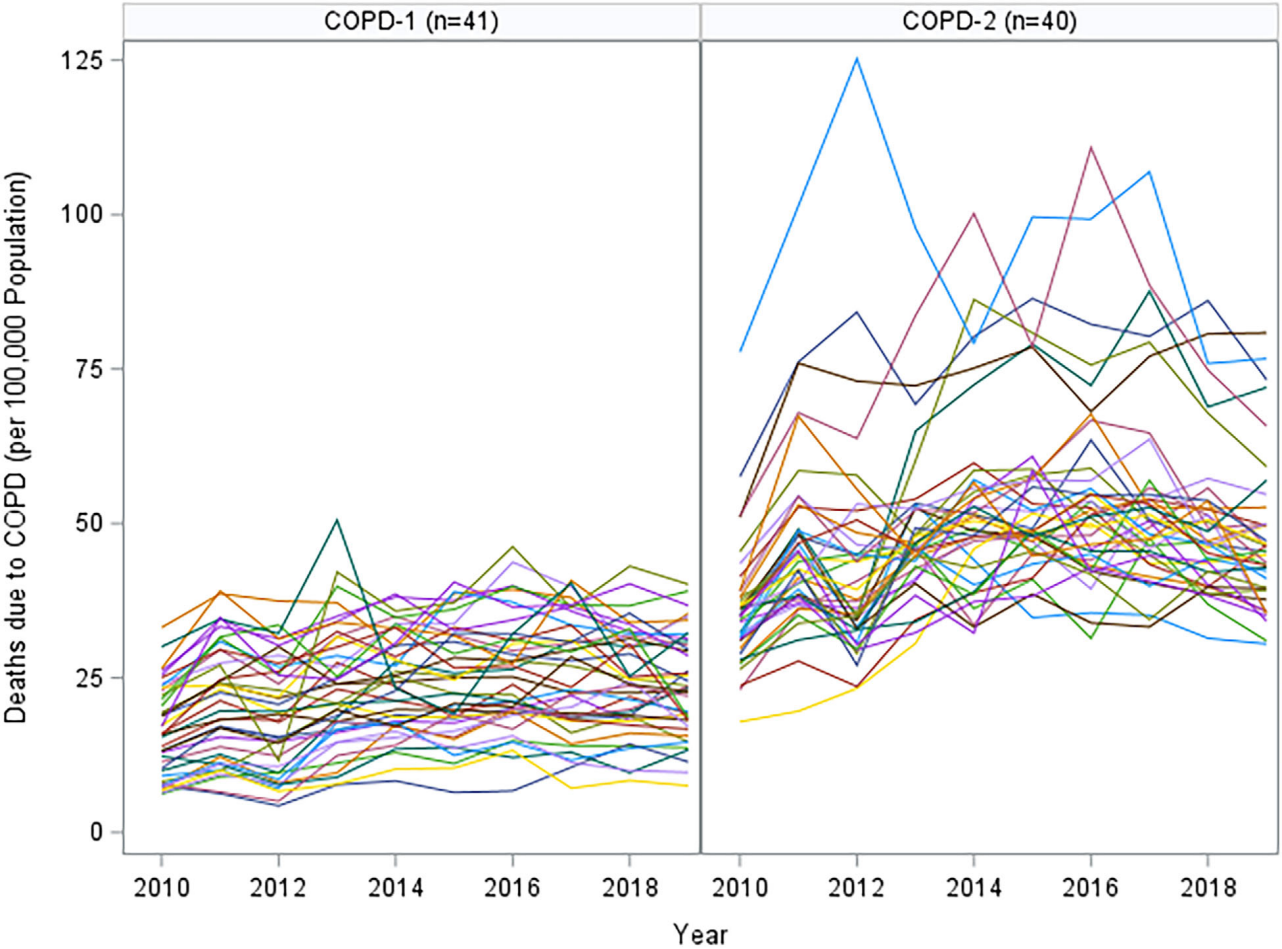
COPD profiles by SAS TRAJ procedure (each line represents the change profile over 10 years). COPD, chronic obstructive pulmonary disease.

**FIGURE 3:**
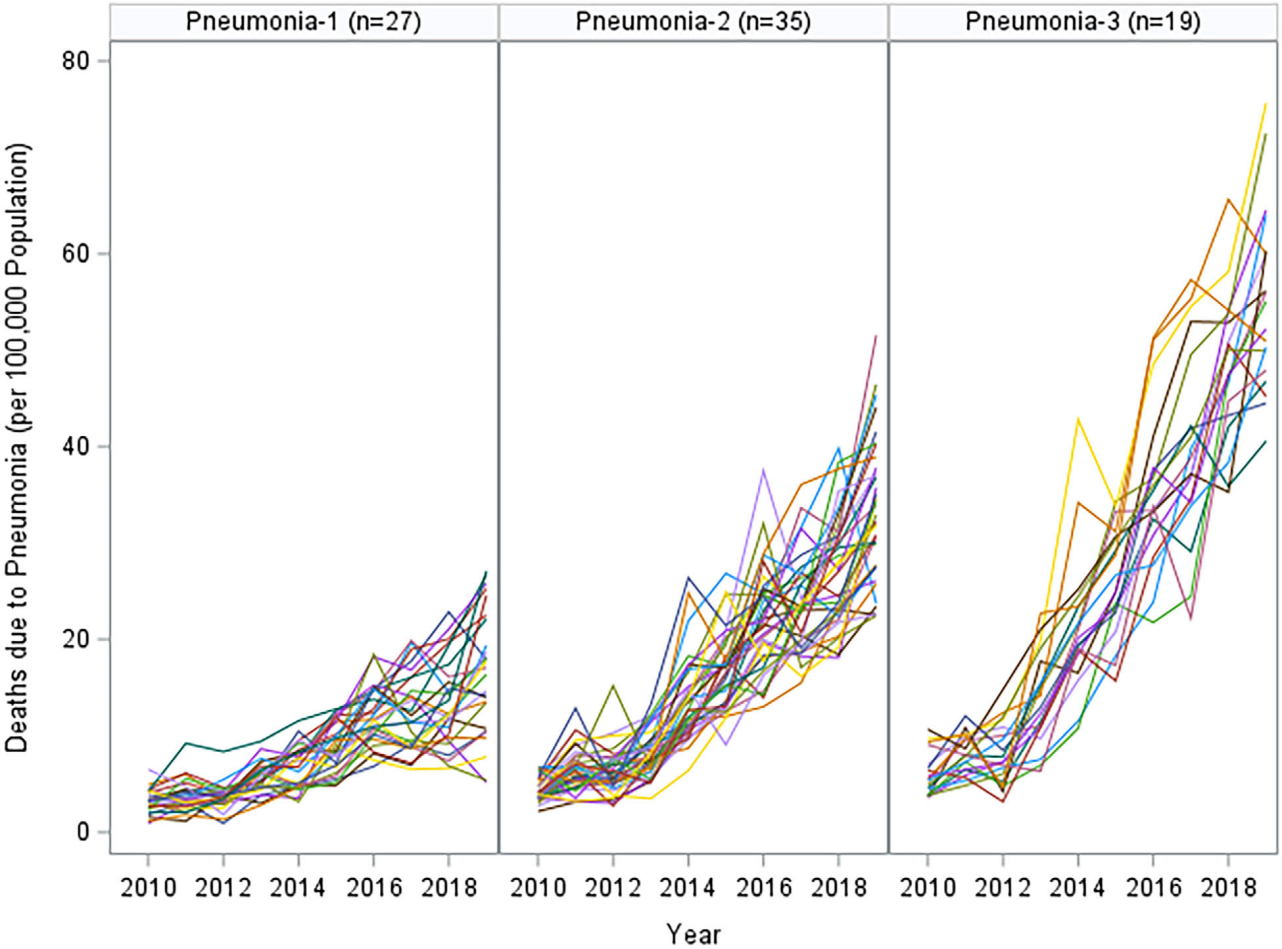
Pneumonia profiles by SAS TRAJ procedure (each line represents the change profile over 10 years).

Our assessment of the longitudinal data of asthma, COPD, and pneumonia through SAS TRAJ procedures resulted in 2, 2, and 3 change‐profiles, respectively. Figures 1, 2, 3 show the actual mortality profiles where each line represents a province and the header includes the number of provinces that fall in a given change trajectory cluster.

For asthma, the data have been divided into 2 groups among the same 81 provinces. Asthma‐1 consists of 52 provinces and demonstrates a relatively stable pattern of death rates, with some fluctuations over time. This stability could indicate effective management of asthma or a low prevalence of severe cases. Asthma‐2, comprising 29 provinces, displays a less stable pattern, with sharp increases at certain points. These spikes may point to intermittent exacerbations of asthma, possibly due to environmental triggers or variations in access to effective asthma care and management.

The COPD death rates are also categorized into two groups. COPD‐1 includes 41 provinces and shows a fluctuating pattern with a broad range of death rates. Although there are upward trends, the group's profile suggests that the situation might be under some degree of control or that there are complex factors influencing mortality rates. COPD‐2, with 40 provinces, is characterized by higher peaks in death rates, indicating episodes of increased mortality. This could be due to environmental factors, healthcare access issues, or other socioeconomic factors impacting COPD management and outcomes.

The longitudinal profile for pneumonia across 81 provinces of Turkiye shows 3 distinct trajectories. Pneumonia‐1, with 27 provinces, indicates a moderate yet consistent upward trend in pneumonia death rates over a 10‐year span. The increases are steady, suggesting a gradual change in factors contributing to pneumonia mortality. Pneumonia‐2, which includes 35 provinces, starts at higher initial death rates than Group 1 and exhibits a more marked upward trend, with greater variability suggesting diverse factors at play across these provinces. Pneumonia‐3, the smallest with 19 provinces, shows the most pronounced increase in death rates. The steep rise in this group indicates that these provinces may be experiencing significant health challenges related to pneumonia.

Tables 1, 2, 3 display the significant predictors identified (markers that were not significant at the FDR of 5% were not reported in these tables). For asthma, none of the predictors passed the 0.05 FDR threshold, and therefore, we presented the potential predictors passing the traditional 0.05 Type‐1 error threshold.

**TABLE 1:** Summary of markers found associated with asthma profiles (CV is coefficient of variance, and SD is standard deviation).

Predictor	OR (95% CI)	*p* value	FDR corrected *p* value
Air pressure (median)	0.99 (0.98, 1.00)	0.0463	0.47
Elderly ratio (age ≥ 65)	1.39 (1.15, 1.68)	0.0005	0.028
Minimum temperature (median)	0.83 (0.73, 0.95)	0.0061	0.20
Particulate matter‐10 (median)	0.96 (0.93, 0.99)	0.0159	0.26

**TABLE 2:** Summary of markers found associated with chronic obstructive pulmonary disease (COPD) profiles (CV is coefficient of variance, and SD is standard deviation).

Predictor	OR (95% CI)	*p* value	FDR corrected *p* value
Elderly ratio (age ≥ 65)	2.27 (1.60, 3.22)	<0.0001	0.0002
Humidity (CV)	0.93 (0.88, 0.98)	0.0037	0.0270
Maximum temperature (median)	0.76 (0.63, 0.93)	0.0069	0.0400
Mean temperature (median)	0.65 (0.51, 0.81)	0.0002	0.0030
Minimum temperature (median)	0.74 (0.64, 0.86)	<0.0001	0.0020
Particulate matter‐10 (SD)	0.89 (0.81, 0.97)	0.0059	0.0380
Rainy days (CV)	0.94 (0.90, 0.97)	0.0013	0.0160
Sun radiation (per 10 units)	0.93 (0.89, 0.98)	0.0028	0.0240
Sunlight (per 100 units)	0.74 (0.62, 0.89)	0.0015	0.0160

**TABLE 3:** Summary of markers found associated with pneumonia profiles (CV is coefficient of variance, and SD is standard deviation).

Predictor	OR (95% CI)	*p* value	FDR corrected *p* value
Air pressure (median)	1.02 (1.01, 1.03)	<0.0001	<0.0001
Elderly ratio (age ≥ 65)	1.86 (1.50, 2.30)	<0.0001	<0.0001
Ever alcohol use	1.11 (1.05, 1.17)	0.0002	0.0008
Humidity (CV)	0.86 (0.81, 0.91)	<0.0001	<0.0001
Humidity (median)	1.12 (1.06, 1.19)	<0.0001	0.0002
Humidity (SD)	0.72 (0.63, 0.82)	<0.0001	<0.0001
Male ratio (%)	0.53 (0.31, 0.89)	0.0158	0.0420
Manual rainy days (CV)	0.96 (0.94, 0.99)	0.0125	0.0360
Manual rainy days (median)	1.25 (1.04, 1.49)	0.0162	0.0420
Maximum temperature (CV)	0.85 (0.80, 0.91)	<0.0001	<0.0001
Maximum temperature (SD)	0.42 (0.30, 0.58)	<0.0001	<0.0001
Mean temperature (CV)	0.97 (0.95, 0.99)	0.0041	0.0140
Mean temperature (SD)	0.23 (0.14, 0.39)	<0.0001	<0.0001
Minimum temperature (SD)	0.23 (0.12, 0.42)	<0.0001	<0.0001
Particulate matter‐10 (CV)	0.95 (0.91, 0.99)	0.0101	0.0330
Particulate matter‐10 (SD)	0.87 (0.80, 0.94)	0.0003	0.0010
Rainy days (CV)	0.93 (0.90, 0.97)	0.0001	0.0006
Rainy days (median)	1.25 (1.05, 1.48)	0.0113	0.0340
Sun radiation (per 10 units)	0.89 (0.85, 0.94)	<0.0001	<0.0001
Sunlight (per 100 units)	0.72 (0.61, 0.85)	0.0001	0.0005

In the context of asthma, we identified only four potential predictors. However, none of these predictors except elderly age proportion passed the FDR threshold of 0.05, implying a need for cautious interpretation. Notably, factors, like air pressure (median), minimum temperature (median), and particulate matter‐10 (median), showed some level of association, as indicated by their respective *p* values (0.0463, 0.0061, and 0.0159). Despite this, their FDR‐corrected *p* values did not meet the stringent criteria, suggesting that these associations might not be robust. Provinces with higher proportion of elderly population were more likely to be in the higher asthma mortality cluster with every 1% increase in the elderly population resulting in about 40% increased odds to be in the higher asthma mortality cluster.

For COPD, the identified predictors displayed a stronger statistical significance. Factors such as humidity (coefficient of variance), maximum temperature (median), mean temperature (median), minimum temperature (median), particulate matter‐10 (SD), and rainy days (coefficient of variance) passed both the traditional *p* value threshold and the more rigorous FDR threshold. The size of elderly age population was a significant predictor of COPD as well. This implies a more reliable association between these environmental factors and COPD profiles.

**FIGURE 4:**
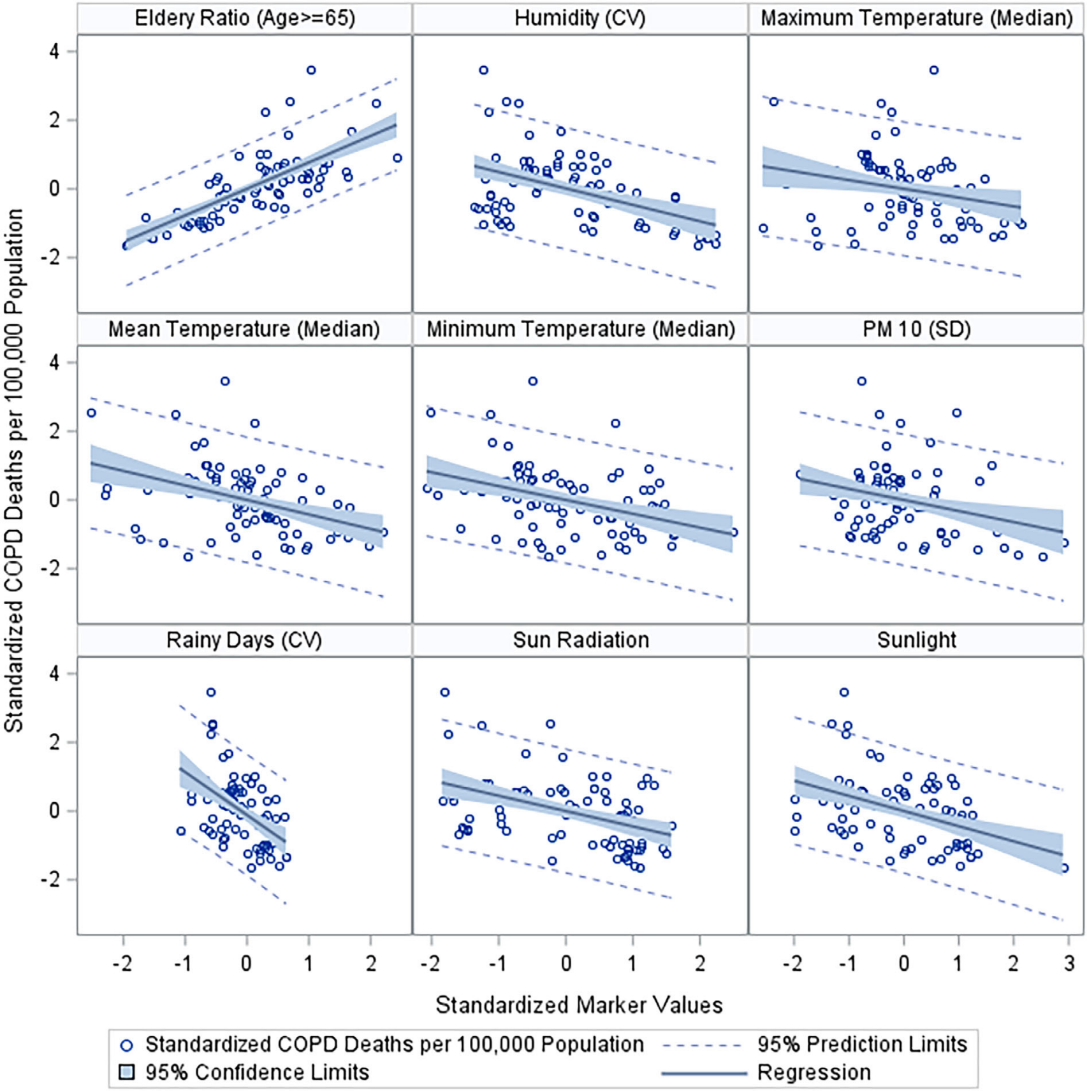
Significant markers indicative of an association with deaths due to COPD. COPD, chronic obstructive pulmonary disease; CV, coefficient of variation; SD, standard of deviation.

**FIGURE 5:**
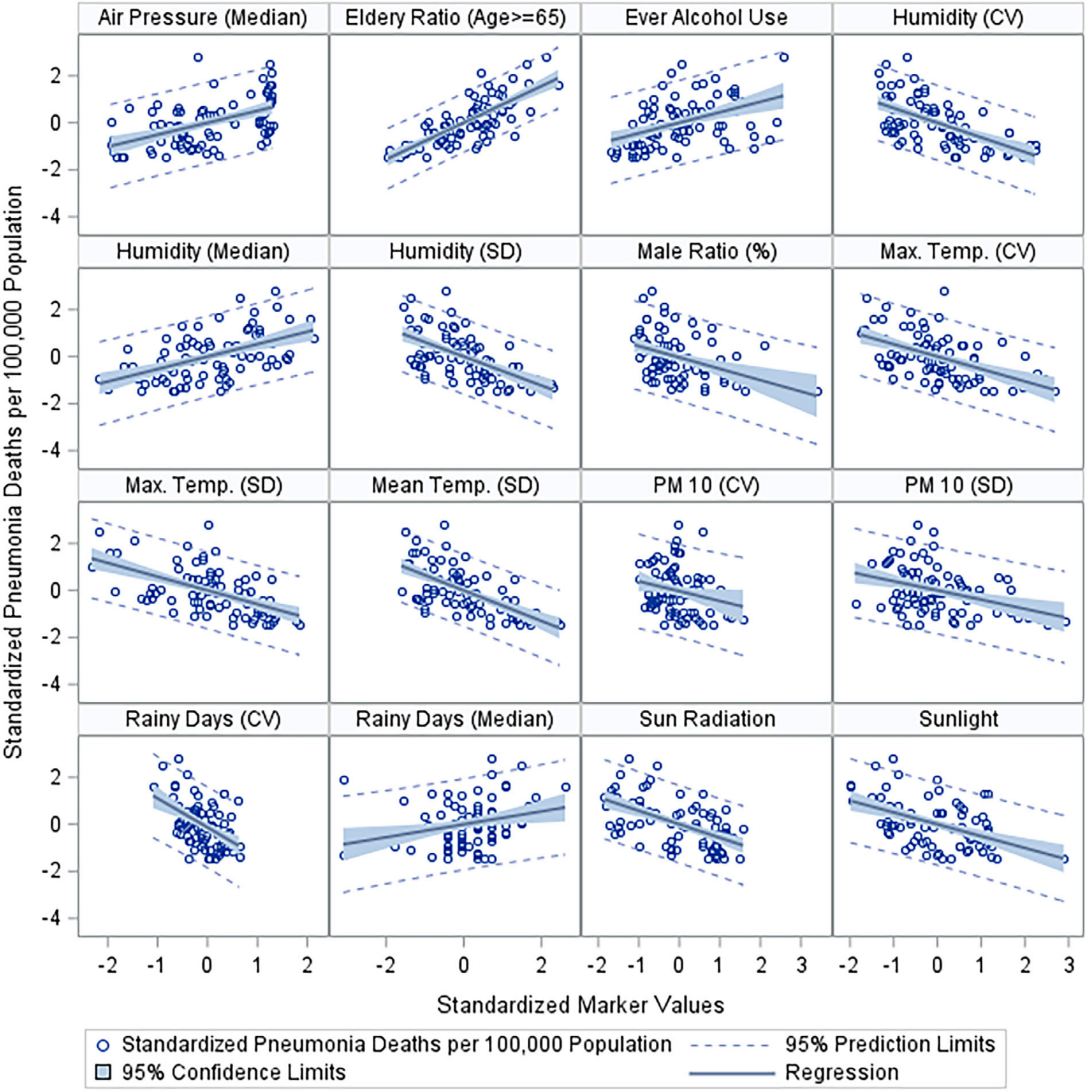
Significant markers indicative of an association with deaths due to pneumonia. CV, coefficient of variation; SD, standard of deviation.

Pneumonia profiles revealed a broad range of significant predictors, with many passing the stringent FDR threshold, including elderly age proportion and male ratio of the provinces. Notable predictors include air pressure (median), ever alcohol use, various measures of humidity (coefficient of variance, median, and SD), manual rainy days (both coefficient of variance and median), maximum temperature (both coefficient of variance and SD), mean temperature (both coefficient of variance and SD), minimum temperature (SD), particulate matter‐10 (both coefficient of variance and SD), and rainy days (both coefficient of variance and median). The significance of these predictors, especially given their FDR‐corrected *p* values, suggests a robust association with pneumonia profiles. We illustrated these associations graphically on the basis of the standardized measures of both the response and the predictor (Figures 4 and 5).

## 4 Discussion

The current longitudinal study provides insights into the death rates from asthma, COPD, and pneumonia across 81 provinces in Turkiye, alongside an examination of associated environmental and socioeconomic predictors.

The trajectories for asthma suggest two distinct patterns among the provinces. Group 1 provinces have shown a consistent pattern, potentially indicative of effective asthma management strategies or lower incidences of severe cases within these regions. This observation aligns with the findings of [16], who note that stability in asthma mortality rates can often reflect successful public health interventions. Group 2 provinces, however, exhibit variability and peaks in asthma death rates, which may correlate with environmental factors or disparities in healthcare access, as discussed by [17].

Despite identifying potential predictors for asthma, such as air pressure and minimum temperature, their lack of significance post‐FDR adjustment calls into question the robustness of these associations. This necessitates further investigation into the complex interplay of genetic, environmental, and healthcare‐related factors contributing to asthma morbidity, as outlined by Tattersfield [18].

The COPD profiles indicate more pronounced variability, with certain provinces experiencing higher mortality peaks. Our analysis identified significant predictors, including humidity and temperature variations, which are corroborated by the works of Lee et al. [19], suggesting that environmental factors play a substantial role in COPD exacerbations. The statistical significance of these predictors, even after FDR correction, strengthens the evidence for their association with COPD mortality.

Moreover, socioeconomic determinants, as well as healthcare accessibility, might be implicated in the observed mortality patterns, resonating with the findings of Mannino and Buist [20], who emphasize the influence of such factors on COPD outcomes.

The analysis of pneumonia mortality revealed three distinct trajectories. The consistent upward trend in Group 1 provinces could reflect changes in population demographics, such as aging, or alterations in the virulence of infectious agents. Group 2's variability and the pronounced increase in Group 3's death rates may indicate disparities in healthcare provision, environmental stressors, or differences in the prevalence of risk factors such as smoking and alcohol use, which have been identified as significant predictors in our study. These associations are particularly noteworthy given their robustness post‐FDR correction, echoing the work of Torres et al. [21], who highlight the multifaceted nature of pneumonia risk factors.

Our graphical illustrations (Figures 4 and 5) underscore the relationships between environmental factors and respiratory disease mortality. These visual representations support the quantitative findings and provide a clear depiction of the potential impact of these predictors. The link between particulate matter and RD, for example, is well established in the literature, with Pope et al. [22] documenting the adverse effects of air pollution on lung health.

The strength of the associations observed, particularly for COPD and pneumonia, underlines the importance of environmental health measures and reinforces the need for targeted interventions. Public health policies aimed at mitigating the impact of environmental pollutants, as suggested by Dockery et al. [23], could be crucial in reducing the burden of these diseases.

### 4.1 Strengths, Weaknesses, and Limitations

One of the main strengths of our study is the use of the SAS TRAJ procedure, which allows for the analysis of longitudinal data to identify distinct change profiles. This method provides a nuanced understanding of trends over time, as seen in our identification of two profiles for asthma and COPD and three for pneumonia.

However, a notable weakness is the reliance on ecological data, which may not account for individual‐level variations and could introduce ecological fallacy. Among the RD, we had data only on COPD, pneumonia, and asthma, although the RD include other restrictive RD such as pulmonary fibrosis and sarcoidosis, other infectious diseases such as tuberculosis, and other pulmonary vascular diseases such as pulmonary hypertension and embolism. In that regard, we are only looking into the part of the respiratory disease platform. Moreover, although we have identified significant predictors for COPD and pneumonia, the lack of robust asthma predictors after FDR correction suggests limitations in the predictive power for this condition. This could be due to the complex nature of asthma, which may be influenced by a multitude of genetic, lifestyle, and environmental factors not fully captured in our study. Another limitation is the potential for misclassification of deaths and the quality of data reporting, which can vary across provinces and over time. This variability can affect the reliability of our results and the observed associations.

The study has significant public health implications. The associations between environmental factors like air quality and temperature with respiratory disease mortality rates underscore the need for public health interventions aimed at environmental protection. Policy‐makers could use these results to prioritize investments in air quality improvements and climate change mitigation strategies. The study highlights the importance of access to healthcare services and the potential impact of socioeconomic factors on disease outcomes. This suggests a need for targeted healthcare services in provinces with higher mortality rates and the implementation of preventative measures, especially for vulnerable populations.

Future research should focus on individual‐level data to confirm the ecological associations found in this study. Longitudinal cohort studies could provide deeper insights into the causative factors behind the observed mortality rates. Additionally, investigating the role of indoor air quality, occupational exposures, and personal lifestyle choices, such as diet and physical activity, could provide a more comprehensive understanding of the risk factors for RD.

There is also a need for studies assessing the effectiveness of public health interventions that have been implemented in response to these findings. This could include evaluating the impact of improved air quality standards and healthcare accessibility on the mortality rates of these diseases. Moreover, future research should consider the potential impacts of global phenomena such as climate change on respiratory health, extending the study to a broader range of environmental factors.

## 5 Conclusion

Our study contributes to the understanding of the epidemiology of RD in Turkiye, highlighting the role of environmental and lifestyle factors as significant predictors of mortality. The robust associations identified for COPD and pneumonia, supported by the literature, emphasize the need for continued research into preventative strategies and interventions to manage these diseases effectively.

## Author Contributions

M.K. generated the research idea, data requests, conducted data analyses, and manuscript preparation. A.N.K. conducted the literature search, background research, and manuscript preparation.

## Ethics Statement

Our research protocol was approved by Istanbul Medipol University Ethics Committee (Application number: 10840098‐604.01.01‐E.53819). The Ethics Committee waived the need for Informed Consent as there is no human subject involved in this research. Data are simply province‐level mortality data provided by the Turkish Statistical Institute per year.

## Consent

The authors have nothing to report.

## Conflicts of Interest

The authors declare no conflicts of interest.

## Code Availability

The authors have nothing to report.

## Data Availability

As the death records data utilized in this report were granted access only to the corresponding author, we do not have the permission to share these data components; however, we can share the environmental data upon request. Dr. Mehmet Kocak can be contacted at mehmetkocak@medipol.edu.tr regarding such data requests.
